# Association of Insomnia with Functional Outcomes Relevant to Daily Behaviors and Sleep-Related Quality of Life among First Nations People in Two Communities in Saskatchewan, Canada

**DOI:** 10.3390/clockssleep6040039

**Published:** 2024-10-12

**Authors:** Chandima P. Karunanayake, James A. Dosman, Najib Ayas, Mark Fenton, Jeremy Seeseequasis, Reynaldo Lindain, Warren Seesequasis, Kathleen McMullin, Meera J. Kachroo, Vivian R. Ramsden, Malcolm King, Sylvia Abonyi, Shelley Kirychuk, Niels Koehncke, Robert Skomro, Punam Pahwa

**Affiliations:** 1 Canadian Centre for Rural and Agricultural Health, University of Saskatchewan, 104 Clinic Place, Saskatoon, SK S7N 2Z4, Canada; james.dosman@usask.ca (J.A.D.); kathleen.mcmullin@usask.ca (K.M.); meera.kachroo@usask.ca (M.J.K.); shelley.kirychuk@usask.ca (S.K.); niels.koehncke@usask.ca (N.K.); pup165@mail.usask.ca (P.P.); 2 Department of Medicine, University of Saskatchewan, Royal University Hospital, 103 Hospital Drive, Saskatoon, SK S7N 0W8, Canada; mef132@mail.usask.ca (M.F.); r.skomro@usask.ca (R.S.); 3 Division of Critical Care Medicine, Department of Medicine, Faculty of Medicine, University of British Columbia, 2775 Laurel Street, Vancouver, BC V5Z 1M9, Canada; nayas@providencehealth.bc.ca; 4 Community B, P.O. Box 250, Montreal Lake, SK S0J 1Y0, Canada; 5 Community A, P.O. Box 96, Duck Lake, SK S0K 1J0, Canada; 6 Department of Community Health & Epidemiology, College of Medicine, University of Saskatchewan, 107 Wiggins Road, Saskatoon, SK S7N 5E5, Canada; malcolm.king@usask.ca (M.K.);; 7 Department of Academic Family Medicine, University of Saskatchewan, West Winds Primary Health Centre, 3311 Fairlight Drive, Saskatoon, SK S7M 3Y5, Canada; viv.ramsden@usask.ca

**Keywords:** insomnia, sleep, functional impairments, FOSQ-10, First Nations

## Abstract

Insomnia is a common sleep complaint in Canada and is associated with increased use of health care services and economic burden. This paper examines the association of insomnia with functional outcomes relevant to daily behaviors and sleep-related quality of life among First Nations participants using the Functional Outcomes of Sleep Questionnaire (FOSQ-10). The First Nations Sleep Health Project follow-up survey was conducted in partnership with two Cree First Nations in the summer of 2022, where 355 individuals participated. Statistical analysis was conducted using logistic regression models. The mean age of the participants was 40.76 ± 14.60 (SD) years, and 59.4% were females. The prevalence of chronic insomnia (Insomnia Severity Index score of ≥15) was 21.0%, with more females (26.1%) than males (13.8%) experiencing it among the 348 participants. Overall, the mean FOSQ-10 score was 17.27 ± 2.98 among the 350 participants, with those who had clinical insomnia reporting significantly lower scores than those without clinical insomnia (mean ± SD: 14.6 ± 3.9 vs. 18.0 ± 2.1; *p* < 0.001). The FOSQ-10 scores indicated sleep-related functional impairment (FOSQ-10 total score < 17.90) in 46.6% of participants. After adjusting for age, excessive daytime sleepiness, sex, and regular use of prescription medication, we found that clinical insomnia was significantly associated with functional impairments. In fact, a person with clinical insomnia was 3.5 times more likely to have functional impairments than those without clinical insomnia. This study highlights the significant association between insomnia and functional impairments related to daily behaviors and quality of life in two First Nation communities. Identifying this association can help healthcare providers to diagnose and treat patients with insomnia in these communities.

## 1. Introduction

Insomnia creates significant clinical impairments in social and work-related activities of life, as evidenced by low work productivity, frequent absenteeism, decreased cognition and mood, as well as increased morbidity of psychological and physical illness. These impairments are associated with increased use of health care services and economic burden on society. In Canada, insomnia is a highly prevalent comorbid condition in the general population. The prevalence of insomnia in the Canadian general population ranges from 13.4% to 38% [1,2,3,4,5,6] and varies by measures and definitions. We previously reported that the prevalence of insomnia in two Saskatchewan First Nation communities was 19.2% based on a baseline study with the same populations [7]. Overall, insomnia is more common in women than in men, and its prevalence increases with age in both sexes [8].

Insomnia significantly impairs quality of life, with impacts on overall psychological and physical well-being [9]. This study examines the association of insomnia with functional outcomes relevant to daily behaviors and sleep-related quality of life among First Nations participants using the Functional Outcomes of Sleep Questionnaire (FOSQ-10) [10].

## 2. Results

**Figure 1:**
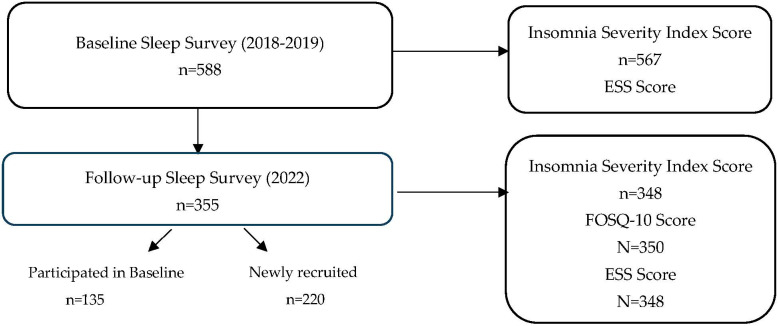
Sleep study baseline and follow-up samples and available scores.

The prevalence of insomnia was 19.2% among participants (*n* = 567), with an Insomnia Severity Index score of ≥15 in the baseline survey conducted in 2018–2019 [7]. The follow-up survey was conducted in both communities in 2022. Three hundred and fifty-five individuals participated in the follow-up survey. Of those, 135 participated in both baseline and follow-up. Two hundred and twenty new participants participated in 2022 (Figure 1).

The FOSQ-10 questionnaire was only included in the follow-up survey. Of the 355 participants, the FOSQ-10 total score was available for 350 participants (Figure 1). Overall, the mean FOSQ-10 total score was 17.27 ± 2.98, lower than the functional impairment cut-off of 17.90 (Table 1). Female FOSQ-10 scores were significantly different from male FOSQ-10 scores (*p* = 0.002) (Table 1). The mean FOSQ-10 scores were significantly lower for females than males. This suggests that females have an increased burden of symptoms due to sleepiness compared with males.

**Figure 2:**
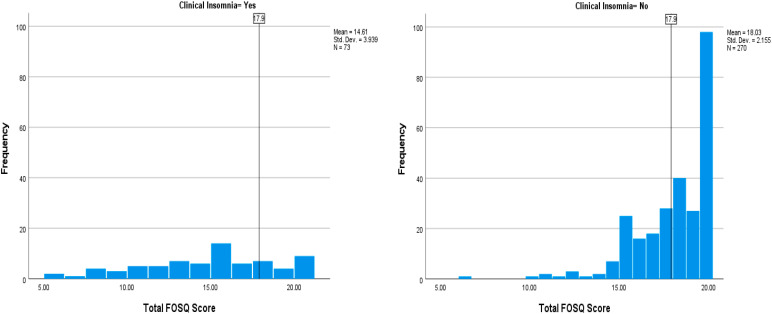
Total FOSQ Score versus Clinical Insomnia Status (Yes/No).

The prevalence of insomnia was 21.0% among participants with an Insomnia Severity Index score of ≥15, with more females (72.6%) than males (27.4%) experiencing it among the 348 participants in the 2022 follow-up survey (Table 2). Participants with clinical insomnia had significantly lower FOSQ 10 scores compared with participants without clinical insomnia (*p* < 0.001). This was confirmed by the individual FOSQ-10 scores (Figure 2), where the total FOSQ-10 score was mostly lower in participants with insomnia.

The prevalence of clinical insomnia was much higher in females (26.1%; 53/203) than in males (13.8%; 20/145). Participants with clinical insomnia had a significantly higher Epworth Sleepiness Scale scores compared with participants without clinical insomnia (*p* < 0.001) (Table 2).

FOSQ-10 scores were available for 350 participants and of those nearly half (46.6%) demonstrated sleep-related functional impairment. Female sex, taking prescription medication, clinical insomnia, excessive daytime sleepiness, and loud snoring were factors associated with functional impairments relevant to daily behaviors and sleep-related quality of life (Table 3). There was also evidence of a difference in the proportion of males and females with FOSQ-10 scores < 17.90 (*p* = 0.002). The proportion of males with FOSQ-10 scores < 17.90 was 36.6% (52/142) compared with 53.4% (111/208) of females. Participants with clinical insomnia reported a higher proportion of functional impairments relevant to daily behaviors and sleep-related quality of life compared with non-impaired participants (34.4% vs. 10.2%). Similarly, participants with abnormal daytime sleepiness reported a higher proportion of functional impairments compared with non-impaired participants (25.8% vs. 14.7%). About one-third of the participants reported loud snoring (30.5%). Of those, more than half (55.2% = 58/105) had a FOSQ-10 total score < 17.90, suggesting they were more likely to have an increased burden of symptoms due to sleepiness.

Adjusting for age, excessive daytime sleepiness, sex, regular use of prescription medication, and loud snoring, the presence of clinical insomnia was significantly associated with functional impairments relevant to daily behaviors and sleep-related quality of life (Table 4). A person with clinical insomnia was 3.5 times more likely to have functional impairments relevant to daily behaviors and sleep-related quality of life. None of the interactions were significant. Women were more likely to report functional impairments relevant to daily behaviors and sleep-related quality of life compared with men. Regular use of prescription medications was also associated with an increased risk of functional impairments.

## 3. Discussion

In the current study, it was observed that nearly half of the study population reported sleep-related functional impairment as measured by FOSQ-10, a tool that assesses sleep-specific health-related quality of life. This study explored the association of insomnia with functional outcomes relevant to daily behaviors and sleep-related quality of life among First Nations participants using the FOSQ-10 questionnaire. The mean FOSQ-10 total scores were lower (indicating more impaired sleep-related quality of life) in the clinical insomnia group compared with those with no clinical insomnia. A greater percentage (74.0%) of participants with clinical insomnia had impaired daily functioning (FOSQ-10 total scores < 17.90) compared with those without clinical insomnia (38.1%). After adjusting for other factors, this study found that a person with clinical insomnia was 3.5 times more likely to have functional impairments relevant to daily behaviors and sleep-related quality of life. Additionally, this study’s findings align with those of Steffen et al., who also reported an association between insomnia and lower FOSQ-10 scores, indicating poorer sleep-related quality of life [11].

According to several studies [3,12,13], clinical insomnia is more prevalent in females than in males. In this study, the scores of the Functional Outcomes of Sleep Questionnaire (FOSQ-10) were significantly lower for females than for males. Females were 1.7 times more likely to experience functional impairments affecting daily behaviors and sleep-related quality of life compared with males. This indicates that females are more likely to suffer from symptoms of sleepiness, as supported by other studies [14,15]. Boccabella et al. found that women were more likely to report increased symptoms of sleepiness compared with men, demonstrated by lower FOSQ-10 scores (*p* < 0.001) [14]. A review of sleep and women’s health revealed that sleep disturbances and sleep disorders are common throughout a woman’s life [16]. Significant biological changes, such as menstruation, pregnancy, and menopause, can lead to impaired sleep quality. Poor sleep can result in tiredness, fatigue, impaired daytime functioning, and mood issues, all of which are crucial to a woman’s quality of life.

Sleep apnea is associated with recurrent airway obstruction during sleep, leading to sleep fragmentation, poorer sleep quality, cognitive dysfunction, and impaired daytime function [17,18]. Loud snoring is a cardinal symptom of sleep apnea, and this is likely a marker of the presence of obstructive sleep apnea (OSA) [17,18]. In the current study, the presence of loud snoring was found to be related to functional impairments that affect daily behaviors and quality of life related to sleep. According to Boccabella et al., more than three-quarters of individuals with partners reported that their snoring kept their partner awake and 48.3% reported that their snoring forced either themselves or their partners out of the room, impacting their relationships and quality of life [14].

This study observed that regular use of prescription medication significantly increased the risk of functional impairments related to daily behaviors and sleep-related quality of life. This could be an indication of a measure of comorbidities, or alternatively, this could also reflect a side effect of the medications. However, this study did not provide a list of specific prescription drugs, which is a limitation. Han et al. reported that among older adults, the increased use of prescription opioids and tranquilizers/sedatives was associated with impairments in performing activities of daily living [19]. Further research is needed to explore this association.

There was no significant difference in self-reported sleep duration between participants who had functional impairment and those with no impairments. Further, in this analysis, differences among short, optimal and long sleep durations were compared. The National Sleep Foundation recommends 7–9 h as the optimal sleep duration for adults [20]. Sleep duration can be categorized into three groups: short sleep duration (<7 h); optimal sleep duration (7–9 h); and long sleep duration (>9 h). Even though there was a slightly large proportion of functional impairment in the short sleep duration group (49.4%) and the long sleep duration group (47.9%) compared with the optimal (7–9 h) sleep duration group (44.2%), there were no statistically significant differences among groups (*p* = 0.726). Further, there was no difference in functional impairment (49.4% vs. 47.9%) between the short and long sleep duration groups (*p* = 0.130). Therefore, no associations between subjective sleep duration and functional impairments were observed. There could be other factors, like cultural, environmental, and behavioral factors, that influence sleep duration. Further studies are needed to confirm this association using objective sleep duration measures.

A study conducted by Morin et al. surveyed 2000 Canadians aged 18 and older about their sleep patterns and found that 13.4% reported experiencing insomnia [3]. In Canada, it is estimated that 23.8% of adults experience nighttime insomnia symptoms [6]. The prevalence of insomnia varies depending on the definition used. According to the definition of the Insomnia Severity Index (ISI), the study reported that the prevalence of insomnia among First Nations people was 19.2% in baseline [7] and 21.0% in follow-up studies, respectively. Insomnia symptoms significantly contribute to the economic burden of illness in Canada [6]. Reducing insomnia symptoms in the First Nations population could potentially benefit the quality of life in these populations.

### Strengths and Limitations

The strengths of this study included the relatively large number of participants and the consideration of various potential factors, such as lifestyle, socio-demographic, and sleep characteristics. This project provides evidence of sleep disorders among First Nation communities and the information obtained from the First Nations Sleep Health Project will help to promote awareness about sleep health among First Nations people and aid in prevention and treatment measures. To our knowledge, this study is the first to investigate functional impairments relevant to daily behaviors and sleep-related quality of life in adults living in two rural Cree First Nation communities in Saskatchewan, Canada.

The data from the questionnaire survey were self-reported, which may have introduced recall bias. Another important point to consider is that the study population was relatively young, with a mean age of 40.76 years (SD = ±14.60 years). The names of the prescription drugs were not available. Additionally, the FOSQ-10 questionnaire was only available for the follow-up study, which made it impossible to examine changes in functional impairments over time. Although this study found associations between several factors and functional impairments, it was not possible to establish causal relationships due to the cross-sectional nature of the data. Another limitation was the lack of objective sleep testing to assess objective sleep duration and presence of sleep apnea.

## 4. Materials and Methods

### 4.1. Study Sample

The follow-up survey of the First Nations Sleep Health Project (FNSHP) was completed between May and October of 2022 in collaboration with two Cree First Nation communities (Community A and Community B) in Saskatchewan, Canada. The baseline survey of the First Nations Sleep Health Project (FNSHP) was completed between July 2018 and December 2019 and details have been published elsewhere [7,21]. The overall goal of the FNSHP was to investigate the relationships between sleep disorders, risk factors, and co-morbidities, and to evaluate local diagnosis and treatment. Ethics approval was obtained from the University of Saskatchewan’s Biomedical Research Ethics Board (Certificate No. Bio #18-110) and adhered to Chapter 9 (Research Involving the First Nations, Inuit, and Metis Peoples of Canada) of the Tri-Council Policy Statement: Ethical Conduct for Research Involving Humans, December 2022 [22]. Individual participants gave their written consent in this research collaboration with the two Saskatchewan First Nation communities.

### 4.2. Data Collection

Research assistants were hired from each community and trained to conduct the follow-up surveys in their respective communities. Adults 18 years and older were invited to the Community Health/Youth Centre to participate in the survey. Data were collected via interviewer-administered questionnaires. The survey collected information on demographic variables, individual and contextual determinants of sleep health, and self-reported height and weight. Demographic information about the participants, including age, sex, body mass index, education level, money left at the end of the month, lifestyle factors, sleeping environment, medical history, and sleep health information, was obtained from the survey questionnaire. In addition to the questions on the baseline survey, questions related to COVID-19 and the FOSQ 10 questionnaire [10] that focused on functional outcomes relevant to daily behaviors and sleep-related quality of life were included in the follow-up survey. This manuscript was based on data from the follow-up survey questionnaire. There were 355 individuals who participated in the follow-up study, including 204 individuals from Community A and 151 individuals from Community B.

#### 4.2.1. Insomnia Severity Index (ISI)

The ISI has seven self-reported questions assessing the nature, severity, and impact of insomnia [23,24,25]. The participants were asked to rate the “current” (that is, the last two weeks) severity of their insomnia problems. The questions measure the severity of sleep onset, sleep maintenance, early morning awakening problems, satisfaction with sleep, interference of sleep difficulties with daily functioning, noticeability of sleep problems by others, and distress caused by sleep difficulties [25]. A 5-point Likert scale was used to rate each question, yielding a total score ranging from 0 to 28. The total score was interpreted as follows: absence of insomnia (0–7), sub-threshold insomnia (8–14), moderate insomnia (15–21), and severe insomnia (22–28) [25]. Clinical insomnia was identified if the score was equal to or greater than 15, i.e., that the ISI score was ≥15 [24].

#### 4.2.2. Epworth Sleepiness Scale (ESS)

The degree of sleepiness was assessed using the ESS. The ESS score ranges from 0 to 24. An ESS score > 10 was considered to be abnormal and indicative of excessive daytime sleepiness [26].

#### 4.2.3. FOSQ-10 Questionnaire

Some people have difficulty performing everyday activities when they feel tired or sleepy. The purpose of the FOSQ questionnaire [27] is to find out if participants generally have difficulty carrying out certain everyday activities because they are too sleepy or tired. The scoring protocol was available from the developer, and permission to use was obtained on 2 March 2022. A shorter 10-item version, the FOSQ-10, was published in 2009, using selected items from each sub-scale of FOSQ-30, and provided the same definition of sleepy and tired [28]. Items for FOSQ-10 are distributed among the same subscales as follows: [1] activity level (3 items); [2] vigilance (3 items); [3] intimacy and sexual relationships (1 item); [4] general productivity (2 items); and [5] social outcomes (1 item). FOSQ-10 has similar validity and reliability to the FOSQ-30 with an internal consistency of α = 0.87 [28]. It is easier to implement in a clinical setting. However, the authors recommended that only the total score for the FOSQ-10 be utilized, rather than individual subscales, because of the limited number of items in each subscale for the FOSQ-10. FOSQ 10 scores ranged from 5–20 points, with higher scores indicating better functional status. An FOSQ total score of less than 17.90 defines ‘functional impairment’ [29]. A cutoff score of less than 17.90 is used for identifying abnormal scores on the FOSQ. This value corresponds to the mean score of a sample of normal individuals who are free of any sleep disorder confirmed by polysomnography [29].

### 4.3. Statistical Analysis

Statistical analyses were conducted using SPSS version 28 ((IBM Corp. Released 2022. IBM SPSS Statistics for Windows, Version 28.0. Armonk, NY, USA: IBM Corp.). Descriptive statistics, mean, median, and standard deviation (SD) were reported for continuous variables, and the *p*-value of the independent samples means *t*-test was reported when comparing the means of samples. For categorical variables, frequency, and percentages (%) were reported.

We used data obtained from the self-reported questionnaire data to determine the association between the outcome of functional impairment and independent variables of interest. Chi-squared tests were used to determine the bivariable association of functional impairment prevalence with independent variables of interest. Multiple logistic regression models were used to predict the relationship between a binary outcome of functional impairment (yes/no) and a set of explanatory variables. A series of logistic regression models were fitted to determine whether potential risk factors, confounders, and interactive effects contributed significantly to the prediction of functional impairment. Based on the bivariable analysis, variables with *p* < 0.20 and less than 25% missing information were candidates for the multiple logistic regression model. All statistically significant variables (*p* < 0.05), as well as important clinical factors (age, sex, daytime sleepiness), were retained in the final multiple regression model. Interactions between potential effect modifiers were examined and were retained in the final model if the *p*-value was <0.05.

## 5. Conclusions

This study emphasizes the significant link between insomnia and impairments in daily functioning and quality of life within two First Nation communities. Recognizing this connection can help healthcare providers to diagnose and treat insomnia with insomnia-specific or other behavioral therapies that may provide an opportunity for improving daytime functioning among First Nations people.

## Figures and Tables

**Table 1:** Mean, standard deviation (SD), and range (minimum–maximum) of FOSQ 10 subscale and total score.

FOSQ 10 Subscale		Questions (Question #)	Possible Range	N	Mean ± SD	Range Min–Max
General Productivity		1. Concentrating 2. Remembering	1–4	340	3.25 ± 0.79	1–4
Activity Level		6. Relations Affected 8. Activity in Morning 9. Activity in Evening	1–4	343	3.37 ± 0.76	1–4
Vigilance		3. Driving short distance 4. Driving long distance 7. Watching Movies	1–4	329	3.54 ± 0.62	1–4
Social Outcomes		5. Visit in their home	1–4	313	3.60 ± 0.69	1–4
Intimacy and Sexual Relationships		10. Desire intimacy	1–4	281	3.52 ± 0.82	1–4
FOSQ 10 Total Score			5–20	350	17.27 ± 2.98	5–20
By Sex:	Male		5–20	142	17.88 ± 2.68	6.5–20
	Female		5–20	208	16.85 ± 3.12 *	5–20

* Female scores were significantly different from male scores (*p* = 0.002).

**Table 2:** Comparison of demographics, Epworth Sleepiness Scale (ESS) score, and FOSQ-10 total score of participants with and without clinical insomnia (*n* = 348).

Variable	with Clinical Insomnia (ISI ≥ 15) Mean ± SD or *n* (%)		without Clinical Insomnia (ISI < 15) Mean ± SD or *n* (%)		*p* Value
Age	*n* = 73	39.63 ± 13.79	N = 274	40.98 ± 14.91	0.485
Sex	*n* = 73	53 (72.6%) females	N = 275	150 (54.5%) females	0.005
Weight in kg	*n* = 69	79.74 ± 18.79	N = 261	82.19 ± 20.93	0.378
Neck Circumference in cm	*n* = 55	37.87 ± 6.11	N = 219	38.45 ± 5.23	0.474
Body Mass Index—BMI (kg/m^2^)	*n* = 67	28.56 ± 6.92	N = 259	28.75 ± 6.92	0.839
Epworth Sleepiness Scale (ESS) Score	*n* = 72	9.75 ± 5.27	N = 273	6.05 ± 4.11	<0.001
FOSQ-10 Total Score	*n* = 73	14. 61 ± 3.94	N = 270	18.03 ± 2.15	<0.001

**Table 3:** Crude association of functional impairment (FOSQ 10 < 17.90) with important factors (results presented in terms of column percentages and unadjusted odds ratios estimates and 95% confidence intervals (CI)) (*n* = 350).

Variable	Total ^#^ *n* (%)	Impaired (FOSQ 10 < 17.90) *n* (%) ^#^	Not Impaired (FOSQ 10 ≥ 17.90) *n* (%) ^#^	*p* Value *	Unadjusted Odds Ratio (95% CI)
All (*n* = 350)		163 (46.6)	187 (53.4)		
Sex (*n* = 350)					
Male	142 (40.6)	52 (31.9)	90 (48.1)	Ref	1.00
Female	208 (59.4)	111 (68.1)	97 (51.9)	0.002	1.98 (1.28, 3.06)
Age, in years (Mean ± SD) (*n* = 349)	40.76 ± 14.60	39.46 ± 14.54	41.89 ± 14.60	0.122	0.99 (0.97, 1.00)
Age group, in years (*n* = 349)					
18–39	175 (50.1)	85 (52.5)	90 (48.1)	Ref	1.00
40–49	67 (19.2)	30 (18.5)	37 (19.8)	0.597	0.86 (0.49, 1.51)
50–59	61 (17.5)	28 (17.3)	33 (17.6)	0.719	0.90 (0.50, 1.61)
60 and older	46 (13.2)	19 (11.7)	27 (14.4)	0.380	0.75 (0.39, 1.44)
Education Level (*n* = 341)					
High school not completed	217 (63.6)	108 (67.1)	109 (60.6)	Ref	1.00
High school or above	124 (36.4)	53 (32.9)	71 (39.4)	0.212	0.75 (0.48, 1.17)
Body mass index (kg/m^2^) (*n* = 329)					
Neither overweight nor obese	120 (36.5)	57 (37.5)	63 (35.6)	Ref	1.00
Overweight	79 (24.0)	37 (24.3)	42 (23.7)	0.927	0.97 (0.55, 1.72)
Obese	130 (39.5)	58 (38.2)	72 (40.7)	0.648	0.89 (0.54, 1.46)
Smoking status (*n* = 343)					
Never smoker	64 (18.7)	28 (17.4)	36 (19.8)	Ref	1.00
Ex-smoker	38 (11.1)	23 (14.3)	15 (8.2)	0.103	1.97 (0.87, 4.46)
Current smoker	241 (70.3)	110 (68.3)	131 (72.0)	0.787	1.08 (0.62, 1.88)
Physical activity—at least three days per week (*n* = 306)					
No	129 (42.2)	68 (46.9)	61 (37.9)	Ref	1.00
Yes	177 (57.8)	77 (53.1)	100 (62.1)	0.112	0.69 (0.44, 1.09)
Prescription medication use on a regular basis (*n* = 348)					
No	207 (59.5)	82 (50.3)	125 (67.6)	Ref	1.00
Yes	141 (40.5)	81 (49.7)	60 (32.4)	0.001	2.06 (1.33, 3.18)
Clinical Insomnia (*n* = 343)					
No	270 (78.7)	103 (65.6)	167 (89.8)	Ref	1.00
Yes	73 (21.3)	54 (34.4)	19 (10.2)	<0.001	4.61 (2.59, 8.21)
Excessive daytime sleepiness (*n* = 343)					
Normal	275 (80.2)	118 (74.2)	157 (85.3)	Ref	1.00
Abnormal	68 (19.8)	41 (25.8)	27 (14.7)	0.011	2.02 (1.18, 3.47)
Loud snoring (*n* = 344)					
No	239 (69.5)	103 (64.0)	136 (74.3)	Ref	
Yes	105 (30.5)	58 (36.0)	47 (25.7)	0.038	1.63 (1.03, 2.59)
Hours of sleep (Mean ± SD), in hours (*n* = 301)	8.03 ± 1.93	7.92 ± 2.07	8.11 ± 1.83	0.421	0.95 (0.85, 1.07)

SD—Standard deviation; ^#^ Column %; * *p* value reported from logistic regression analysis.

**Table 4:** Adjusted prevalence of functional impairment (FOSQ 10 < 17.90) with important factors (results presented in terms of adjusted odds ratios and 95% CI).

Variable	Adjusted Odds Ratio (95% CI)	*p* Value
Sex		
Male	1.00	
Female	1.69 (1.04, 2.75)	0.033
Age group, in years		
18–39	1.00	
40–49	0.62 (0.31, 1.21)	0.159
50–59	0.59 (0.30, 1.17)	0.130
60 and older	0.48 (0.22, 1.06)	0.068
Prescription medication use on a regular basis		
No	1.00	
Yes	2.17 (1.27, 3.72)	0.005
Clinical Insomnia		
No	1.00	
Yes	3.51 (1.89, 6.52)	<0.001
Excessive daytime sleepiness		
Normal	1.00	
Abnormal	1.40 (0.76, 2.58)	0.287
Loud snoring		
No	1.00	
Yes	1.73 (1.03, 2.89)	0.038

## Data Availability

The First Nations community own and control the data and the data release as per the research agreements with the communities and the OCAP principles (https://fnigc.ca/ocap-training/) (accessed on 16 July 2024). Requests for data access can be made to the Chief and Council of community A at reception@beardysband.com and to the Health Director of community B at director@wchealth.ca.

## Abbreviations

FOSQ	Functional Outcomes of Sleep Questionnaire
FNSHP	First Nations Sleep Health Project
BMI	Body Mass Index
SPSS	Statistical Package for the Social Sciences
SD	Standard Deviation
OR	Odds Ratio
CI	Confidence Interval
ISI	Insomnia Severity Index
ESS	Epworth Sleepiness Scale
